# Temporomandibular disorders cases with high-impact pain are more likely to experience short-term pain fluctuations

**DOI:** 10.1038/s41598-022-05598-w

**Published:** 2022-01-31

**Authors:** Alberto Herrero Babiloni, Fernando G. Exposto, Connor M. Peck, Bruce R. Lindgren, Marc O. Martel, Christophe Lenglet, David A. Bereiter, Lynn E. Eberly, Estephan J. Moana-Filho

**Affiliations:** 1 Division of Experimental Medicine, McGill University, Montreal, QC Canada; 2 Center for Advanced Research in Sleep Medicine, Research Centre, Hôpital du Sacré-Coeur de Montréal (CIUSSS du Nord de-L’Île-de-Montréal), Montreal, QC Canada; 3 Section of Orofacial Pain and Jaw Function, Department of Dentistry and Oral Health, Aarhus University, Aarhus, Denmark; 4 Scandinavian Center for Orofacial Neurosciences (SCON), Aarhus, Denmark; 5 Division of TMD and Orofacial Pain, University of Minnesota School of Dentistry, Minneapolis, MN USA; 6 Masonic Cancer Center, University of Minnesota, Minneapolis, MN USA; 7 Faculty of Dentistry and Department of Anesthesia, McGill University, Montreal, QC Canada; 8 Department of Radiology, University of Minnesota School of Medicine, Minneapolis, MN USA; 9 Center for Magnetic Resonance Research, University of Minnesota, 6-320d Moos Tower, 515 Delaware St. SE, Minneapolis, MN 55455 USA; 10 Department of Diagnostic and Biological Sciences, University of Minnesota School of Dentistry, Minneapolis, MN USA; 11 Division of Biostatistics, School of Public Health, University of Minnesota, Minneapolis, MN USA

**Keywords:** Pain, Chronic pain

## Abstract

Temporomandibular disorders (TMD) patients can present clinically significant jaw pain fluctuations which can be debilitating and lead to poor global health. The Graded Chronic Pain Scale evaluates pain-related disability and its dichotomous grading (high/low impact pain) can determine patient care pathways and in general high-impact pain patients have worse treatment outcomes. Individuals with low-impact TMD pain are thought to have better psychosocial functioning, more favorable disease course, and better ability to control pain, while individuals with high-impact pain can present with higher levels of physical and psychological symptoms. Thereby, there is reason to believe that individuals with low- and high-impact TMD pain could experience different pain trajectories over time. Our primary objective was to determine if short-term jaw pain fluctuations serve as a clinical marker for the impact status of TMD pain. To this end, we estimated the association between high/low impact pain status and jaw pain fluctuations over three visits (≤ 21-day-period) in 30 TMD cases. Secondarily, we measured the association between jaw pain intensity and pressure pain thresholds (PPT) over the face and hand, the latter measurements compared to matched pain-free controls (n = 17). Jaw pain fluctuations were more frequent among high-impact pain cases (n = 15) than low-impact pain cases (n = 15) (OR 5.5; 95% CI 1.2, 26.4; *p* value = 0.033). Jaw pain ratings were not associated with PPT ratings (*p* value > 0.220), suggesting different mechanisms for clinical versus experimental pain. Results from this proof-of-concept study suggest that targeted treatments to reduce short-term pain fluctuations in high-impact TMD pain is a potential strategy to achieve improved patient perception of clinical pain management outcomes.

## Introduction

Chronic pain patients often experience different pain trajectories over time^1,2^. While some individuals report stable pain patterns, others describe wax and wane fluctuations^3,4^. Clinically significant pain fluctuations are disruptive^5^ and more debilitating than momentary pain intensity^2,6,7^, as they result in a sense of lack of control in the patient's life^7,8^. Indeed, pain fluctuations are associated with higher levels of depression and poor mental health^3,6,9,10^, catastrophizing^7,11^, and reduced work efficiency^2^.

It has been reported that patients with painful temporomandibular disorders (TMD), a common chronic orofacial pain condition affecting the temporomandibular joint and/or muscles of mastication^12,13^, also present pain fluctuations over time^14,15^. Clinically significant pain fluctuations, often defined as changes in pain intensity of at least 20 in a 0–100 pain scale^16,17^, have been identified in TMD case samples in both short-^14,14^ and long-term^19,20^ periods. These pain fluctuations are influenced by several factors such as menstrual cycle^15,21^, weather^14^, stress^22^ or treatment modality^22,23^. While the literature related to pain fluctuations and their determinants is comprehensive for several painful conditions such as rheumatoid arthritis^24,25^, osteoarthritis^4,26,27^, and low back pain^10,28^, our current understanding of chronic TMD pain fluctuations is limited. Understanding the factors that render TMD pain patients at greater risk for clinically significant pain fluctuations and treatment strategies to reduce them may lead to improved quality of life for this patient population.

The Graded Chronic Pain Scale (GCPS) is a widely used instrument to assess pain disability level^29^, and its dichotomous grading (high/low impact pain)^30^ can be used to determine patient care strategies and resource allocations^31,32^, while also characterizing individuals with different psychosocial subtypes^33^. Indeed, individuals with low-impact TMD pain are thought to have better psychosocial functioning, more favorable disease course^34^, and better ability to control pain, whereas individuals with high-impact pain present with higher levels of physical and psychological symptoms^33^. Together, these findings suggest that individuals with low- and high-impact TMD pain experience diverse pain trajectories.

Chronic TMD pain involves both peripheral and central mechanisms^35,36^, the latter being suggested as predominant in high-impact pain patients^32,37^. The differential contribution of peripheral and central mechanisms to pain fluctuations in TMD patients is unexplored, and it is possible that clinically significant pain fluctuations are associated with altered quantitative sensory testing (QST) measures that are assumed to assess the peripheral component of the somatosensory system^38^, such as pressure pain thresholds (PPT). Pressure algometry is considered a reliable, clinically accessible technique to assess bodily deep tissue pain sensitivity^38,39^, including orofacial structures^40,41^.

Experimental pain assessed with PPT fluctuates along long-term periods (3–6 months) over the clinical course of painful TMD^42^. By contrast, PPT fluctuations over shorter periods of time (≤ 3 months) were not correlated with clinical pain ratings while TMD patients underwent conservative treatment^43^. Neither of the studies investigated if pain impact level was associated with TMD pain fluctuations. Therefore, the association between experimental pain as assessed with PPT and clinical TMD pain ratings in patients categorized by pain impact status remains to be determined.

Our primary objective was to determine if jaw pain fluctuations over three visits in a ≤ 21 day-period serve as a clinical marker of low- or high-impact TMD pain. Secondarily, in order to examine the possible contribution of experimental pain sensitivity to jaw pain fluctuations over time, we estimated the association between jaw pain intensity ratings and PPT over the face and hand in the same period. PPT measures were also compared to matched pain-free controls to have a reference for the normal variability of PPT over the three visits.

## Methods

### Participants

Data reported here were collected as part of a parent study conducted at the University of Minnesota School of Dentistry (UMN SOD) assessing somatosensory characteristics and neuroimaging outcomes from participants with chronic TMD pain and pain-free controls^44,45^. Of the 52 female participants enrolled in the parent study, five had partial data missing which prevented them to be considered for the present study. Thus, a total of 30 females with chronic TMD pain and 17 age-matched pain‐free controls are included. As in other studies of painful TMD^46–48^, only female participants were recruited due to the significantly greater prevalence of TMD in females^49^. Inclusion criteria for chronic TMD pain cases were: (a) adults (≥ 18 years of age); (b) fulfilment of the diagnostic criteria for temporomandibular disorders (DC/TMD) for myalgia (defined as “pain of muscle origin that is affected by jaw movement, function or parafunction, and replication of this pain occurs with provocation testing of the masticatory muscles”) with or without concurrent arthralgia (defined as “pain of joint origin that is affected by jaw movement, function or parafunction, and replication of this pain occurs with provocation testing of the temporomandibular joint (TMJ)”)^12^. Specifically, pain location for myalgia should be present over the masseter and/or temporalis muscles area. The presence of concurrent pain over the pre‐auricular area (TMJ) was accepted; (c) pain present for a minimum of 6 months and ≥ 15 days of pain in the previous 30 days.

Age‐ matched pain‐free controls were included if they did not report the presence of any persistent pain condition in the previous 6 months and did not meet the DC/TMD criteria for any of the most common pain‐related TMD (myalgia, arthralgia, headache attributed to TMD). Exclusion criteria for all participants were: (a) current acute pain medication use (e.g., opioids, ibuprofen, acetaminophen) that could not be stopped 24 h prior to testing; (b) conditions/diseases associated with altered pain perception: neurological (e.g., multiple sclerosis, trigeminal neuralgia), major psychiatric disorders, diabetes, neoplasm and cardiovascular disorders; (c) substance abuse; and (d) pregnancy.

### Experimental protocol

The study protocol was approved by the UMN Institutional Review Board, and all methods were performed in accordance with the relevant guidelines and regulations. All participants were informed in detail about the experimental protocol and gave oral and written informed consent before entering the study. Full description of the experimental protocol can be found elsewhere^44,45^. Briefly, data were collected during three experimental visits, each of them separated by a 2–4 day period which is defined here as “short-term”. In the first visit, participants completed questionnaires assessing several sociodemographic and anthropometric characteristics. In the same visit, all participants underwent a clinical examination in accordance with the DC/TMD criteria^12^ performed by a trained and calibrated examiner. The two additional experimental visits included quantitative sensory testing and neuroimaging data collection, which were not part of the present study and are not reported further.

Repeated measures were collected at each of the three visits. They included PPT of the face (average PPT of bilateral masseter muscles) and dominant hand (thenar eminence), current jaw pain intensity ratings using a 0–100 numerical rating scale (NRS) and potential confounders for sensory testing (e.g., time of the testing, caffeine intake, medication intake, menstrual cycle).

### Sociodemographic, anthropometric and pain characteristics

Most of the sociodemographic data were collected using the DC/TMD Patient History Questionnaire^12^. As described previously^44,45^, anthropometric characteristics such as age, height and weight were based on self‐report. Pain characteristics encompassed characteristic pain intensity (0–100) from the graded chronic pain scale (GCPS)^29^, pain duration since it was first noticed by the patient, total number of body sites (0–45) marked as painful in the last 30 days using a body drawing^12^ and a comorbidity index based on self‐report for the presence of 18 comorbid conditions (migraine headache, tension-type headache, chronic fatigue syndrome, chronic pelvic pain, chronic low‐back pain, vulvodynia, vulvar vestibulitis syndrome, irritable bowel syndrome, interstitial cystitis, premenstrual dysphoric disorder, neuropathic pain, osteoarthritis, rheumatoid arthritis, obstructive sleep apnea, restless leg syndrome, tinnitus, whiplash and post‐traumatic stress disorder)^44,45^, in a similar way than other studies aiming to describe comorbid conditions in individuals with TMD^13,50^.

### Psychosocial characteristics

To collect psychosocial variables, the following valid and reliable instruments from the comprehensive DC/TMD Axis II^12^ were used; Jaw Function Limitation Scale 20‐items (JFLS-20) for jaw limitation; Patient Health Questionnaire‐9 (PHQ‐9) for depression; Generalized Anxiety Disorder‐7 (GAD‐7) for anxiety; Patient Health Questionnaire‐15 (PHQ‐15) for physical symptoms/somatization; Oral Behavior Checklist (OBC) for oral parafunctions. Additional questionnaires included the Perceived Stress Scale (PSS) for perceived levels of stress^51^ and Pittsburgh Sleep Quality Index (PSQI) for sleep quality^52^. The order of questionnaire administration was randomised for each participant to reduce priming effects^53^ and response bias, e.g., due to fatigue or boredom.

### Experimental pain measures

Participants were seated in a comfortable armchair, in a quiet room with an ambient temperature of approximately 23 °C. Sensory testing was performed using a digital pressure algometer (SOMEDIC, Sweden) fitted with a probe (1 cm^2^ surface area) to deliver blunt pressure with a ramp rate of 50 kPa/s over the thenar eminence of the dominant hand (extra-trigeminal location) and the masseter muscle body (centered within its antero-posterior and supero-inferior dimensions, trigeminal location) in each side. PPT was defined as the pressure in kPa delivered by the pressure algometer which the participants first perceived as painful. Three measurements were taken in each body site and the mean was defined as the PPT for that site. To determine PPT in the face, PPT values of both masseter muscles were averaged, as in previous studies^38,44^. The pressure algometer was calibrated and maintained periodically according to the manufacturer’s instructions.

Confounders that could influence pain sensitivity responses were recorded^54^, including starting time for each visit, first day of menses’ onset (if occurring) and caffeine and medications intake in the last 24 h prior to each of the three visits. If occurring, the menstrual cycle phase was determined as previously described^55^ into the following categories: menstrual, follicular, periovulatory, luteal and premenstrual. Caffeine intake in the last 24 h was divided into three categories: low (< 100 mg/day), moderate (101–200 mg/day) or high (> 201 mg/day)^56^. Participants were asked to not take any acute pain medications 24 h before sensory testing (e.g., opioids, ibuprofen, acetaminophen) and all other pain medications taken in the last 24 h were recorded to derive a score based on the Medication Quantification Scale (MQS)^57^.

### Data reduction

#### Dichotomization using the GCPS

The GCPS scoring algorithm allows classification on a 5-point ordinal scale from grade 0 (pain free) to grade IV (high disability, severely limiting). A dichotomized GCPS grade categorization (low: 0–IIa; high: IIb–IV) was calculated for each TMD case to assign them to one of two groups: high-impact (n = 15) or low-impact (n = 15) pain. This method of GCPS score dichotomization has been used in several studies^30,32,37,58,59^, as it assesses pain intensity and disability by averaging three questions for each with scores ranging from 0 to 10. These scores are then combined with the number of days patients report being prevented from their usual activities in the past 6 months, where high-impact chronic pain could then be aligned with other operational definitions such proposed by the National Pain Strategy, where: “High-impact chronic pain is associated with substantial restriction of participation in work, social, and self-care activities for six months or more”^60^. As a matter of fact, in this study, the 180 days version of the GCPS was used^29^. While this version may sustain more pronounced recall bias than shorter versions (i.e., 30 days for obvious reasons), it possibly captures the impact of pain in a wider manner, possibly being more representative of the “usual” pain of the individual.

#### Clinically significant pain fluctuations

Jaw pain intensity changes of magnitude ≥ 20 in a 0–100 NRS occurring between sequential visits (period 1: visit 1 to visit 2; period 2: visit 2 to visit 3) were considered as positive for pain fluctuations^17^. Given our focus on short periods of time (2–4 days), fluctuations between visit 1 and visit 3 were not considered. The presence of pain fluctuations for each participant in either period was set as a binary outcome (yes/no). Participants who had pain fluctuations in one or both time periods were categorized as having pain fluctuations, whereas participants who did not experience fluctuation in either period were categorized as not having pain fluctuations.

### Statistical analyses

Anthropometric and sociodemographic characteristics collected at visit 1 are presented as means and standard deviations (SD) for continuous variables. Descriptive statistics determined that these data were not normally distributed. Thus, statistical comparison for these characteristics between two groups were conducted using the Mann–Whitney *U* test, while three group comparisons were conducted using the Kruskal–Wallis test followed by post-hoc pairwise comparisons. Bonferroni correction for multiple comparisons was done for the main between-group comparisons.

Before conducting analyses for the primary objective, we first conducted exploratory analyses examining the potential confounding influence of patient demographics, psychosocial and clinical characteristics. Variables that were significantly associated with main study outcomes (i.e., jaw pain fluctuations) were retained as covariates in subsequent analyses.

For our primary objective, Pearson’s Chi Square test was used to assess differences in number of cases experiencing pain fluctuations by TMD group. Binary logistic regression was used to calculate the likelihood of TMD cases experiencing pain fluctuations between visits. In this analysis, the presence of pain fluctuations (yes/no) was considered as a dependent variable, while the TMD group category (low-/high-impact pain) was considered as the independent variable. The results of the logistic regression model are presented as odds ratios (ORs) plus 95% confidence intervals (CI). The alpha threshold for statistical significance was set at 0.05.

For the secondary objective, we assessed within- and between-group differences in current jaw pain ratings (high- and low-impact TMD groups) and PPT (high- and low-impact TMD and pain-free controls groups) in the face and in the hand over the three visits. Thus, a linear mixed model for each outcome was performed assuming an auto regressive variance/covariance matrix for time (assuming adjacent time points are more correlated than non-adjacent time points). Main effects for “group” and “visit”, and the interaction between “group” and “visit” were included. Correlations between jaw pain intensity and PPT of the face and hand for the three visits was also assessed using Pearson correlation analyses and linear models. Finally, we assessed the association of jaw pain intensity ratings and PPT for face and hand with potential sensory testing confounders collected at each visit: starting time of the visit (“6:00 to 10:00”, “10:01 to 14:00”, or “14:01 and later”), menstrual cycle phase (no menses, menstrual, follicular, periovulatory, luteal, premenstrual), caffeine intake (none, low, moderate, high) or medication intake (MQS score).

All statistical analyses were performed with R software version 4.0.2 (R Foundation for Statistical Computing, Vienna, Austria) and SAS version 9.4 (SAS Institute, Inc., Cary, NC, USA).

## Results

Clinical and psychosocial characteristics are reported in Table 1. While age and body mass index were similar between groups (*p* value = 1.000), both groups of TMD cases differed from controls in several clinical and psychosocial characteristics, suggesting higher psychosocial distress for TMD cases regardless of pain impact status. This difference was more evident between the high-impact TMD pain group and pain-free controls, where greater scores were observed across all domains in the high-impact TMD group, including total number painful body sites and number of comorbidities, jaw functional limitation, depressive symptoms and somatization, oral habits and sleep quality (Table 1). However, no significant differences were observed in anxiety or stress. In the low-impact TMD pain group, the total number of painful body sites, jaw functional limitation, oral behaviors, and somatization scores were significantly higher than pain-free controls. Nonetheless, no significant differences were found between high- and low-impact TMD pain groups for any of those measures except characteristic pain intensity (CPI, *p* value = 0.013), which was partially expected as it is included in the algorithm to determine GCPS grades 0 to IIb (grades III and IV are determined from other information, regardless of the CPI).

**Table 1:** Clinical and demographic characteristics collected at visit 1.

	Controls (n = 17)	Low-impact TMD (n = 15)	High-impact TMD (n = 15)	3-Group *p* value	Post-hoc pairwise comparison *p* values		
					Controls versus high-impact TMD	Controls versus low-impact TMD	High- versus low-impact TMD
Age (years)	34.5 ± 13.7	33.9 ± 13.6	33.8 ± 12.6	1.000	–	–	–
BMI (kg/m^2^)	25.7 ± 6.5	26.5 ± 6.3	25.7 ± 7.1	1.000	–	–	–
Characteristic pain intensity (0–100)*	–	47.3 ± 16.1	65.6 ± 10.1	**0.013**	–	–	–
Pain duration (years)	–	14.1 ± 12.1	11.0 ± 8.6	1.000	–	–	–
Total number painful body sites (0–45)	0.5 ± 1.2	13.3 ± 7.9	14.7 ± 9.1	**0.013**	**0.013**	**0.013**	1.000
Comorbidity Index (0–18)	0.4 ± 1.0	2.3 ± 2.0	2.5 ± 1.9	**0.013**	**0.026**	**0.004**	1.000
JFLS‐20 (0–10)	0.0 ± 0.0	1.9 ± 1.0	2.9 ± 1.6	**0.013**	**0.013**	**0.013**	1.000
PHQ‐9 (0–27)	1.8 ± 1.5	6.7 ± 5.4	6.8 ± 5.2	**0.013**	**0.026**	0.065	1.000
GAD‐7 (0–21)	3.1 ± 3.8	6.1 ± 4.1	7.8 ± 6.2	0.195	0.299	0.897	1.000
PHQ‐15 (0–30)	3.7 ± 2.7	8.8 ± 4.3	10.6 ± 6.7	**0.013**	**0.013**	**0.026**	1.000
OBC (0–84)	18.9 ± 7.0	35.3 ± 10.7	35.9 ± 15.7	**0.013**	**0.026**	**0.013**	1.000
PSS (0–40)	10.7 ± 6.4	15.0 ± 6.4	17.3 ± 6.6	0.234	0.247	1.000	1.000
PSQI (0–21)	4.0 ± 2.4	8.2 ± 4.6	9.3 ± 3.4	**0.013**	**0.013**	0.169	1.000

Results are reported as means and standard deviations. Two group comparisons were conducted using Mann–Whitney *U* test, while three group comparisons were conducted using Kruskal–Wallis test. *p* values presented for the main between-group comparisons are Bonferroni corrected for multiple comparisons.

*BMI* body mass index, *JFLS* jaw functional limitation scale; *PHQ* pain health questionnaire, *OBC* oral behavioral checklist; *PSS* perceived stress scale, *PSQI* Pittsburgh sleep quality index.

*Characteristic pain intensity was measured as part of the graded chronic pain scale scoring algorithm.

### Jaw pain fluctuations at the individual level

**Figure 1:**
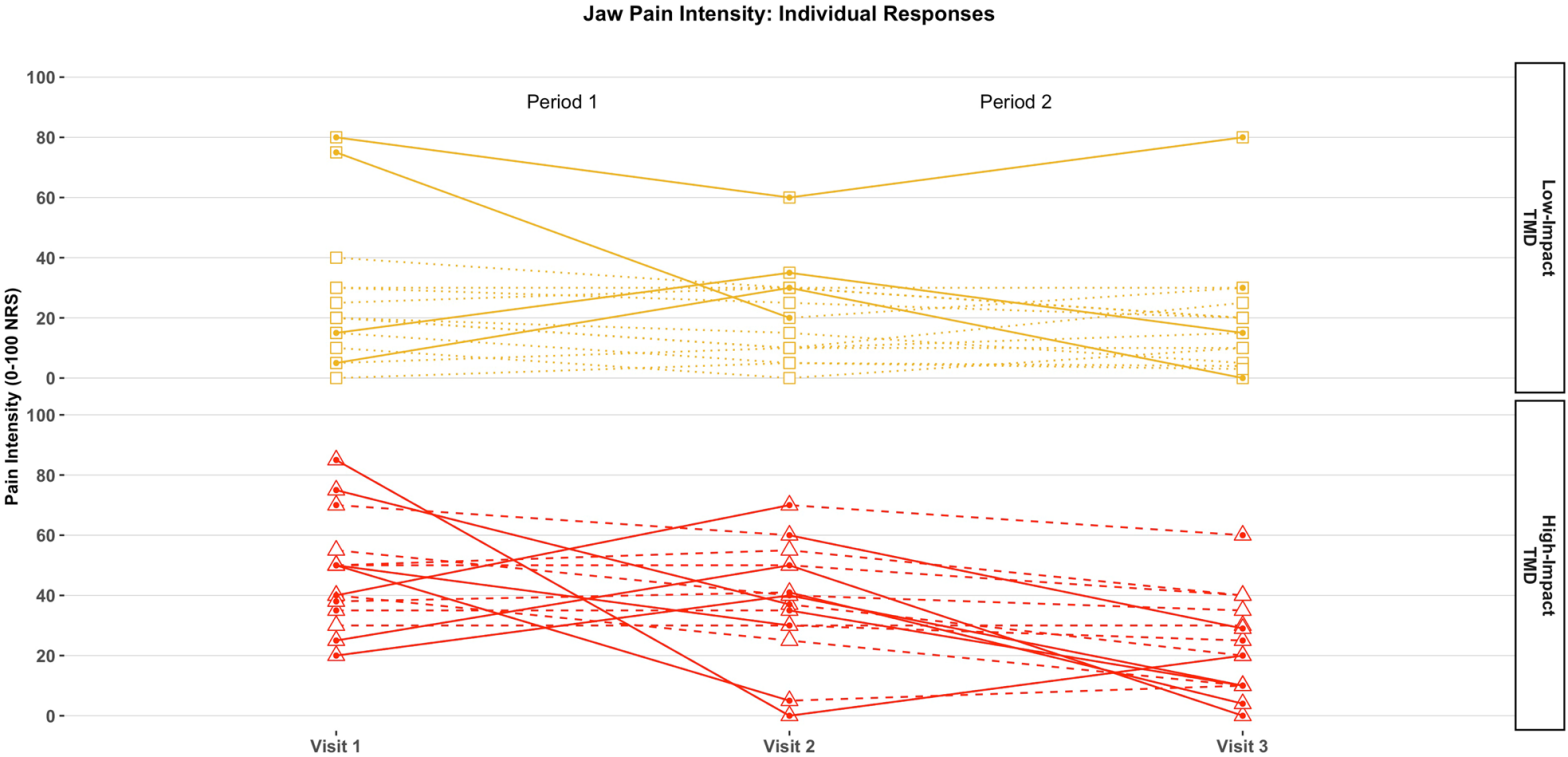
Current jaw pain intensity over time: individual responses. Dotted symbols and solid lines in either one or both periods identify participants who presented with pain fluctuations, defined as jaw pain rating difference ≥ 20 (0–100 NRS) between visit 1 and visit 2 (period 1) or between visit 2 and visit 3 (period 2). Empty symbols and dashed lines in both periods identify participants with no such pain fluctuations.

Jaw pain fluctuations were observed in 14 out of 30 TMD cases, being more frequent among high-impact TMD cases: 10 out of 15 (66.7%), than those with low disability: 4 out of 15 (26.7%); Chi square (df = 1) = 4.53; *p* = 0.033 (Fig. 1). Results from the logistic regression revealed that high-impact TMD cases had more than five times higher odds of experiencing pain fluctuations than low-impact TMD cases (OR 5.5; 95% CI 1.2, 26.4). Exploratory univariate analyses did not reveal any potential variable significantly associated with pain fluctuations, except for a positive trend in the association between age and pain fluctuations when used alone in the model (*p* = 0.079). Based on the importance of age in pain fluctuations as previously reported^61,62^, we decided to include it as a covariate in the logistic regression model which led to higher odds of pain fluctuations in the high-impact compared to low-impact TMD group (OR 7.4; 95% CI 1.2, 43.5; *p* value = 0.028).

### Jaw pain mean ratings over time

**Figure 2:**
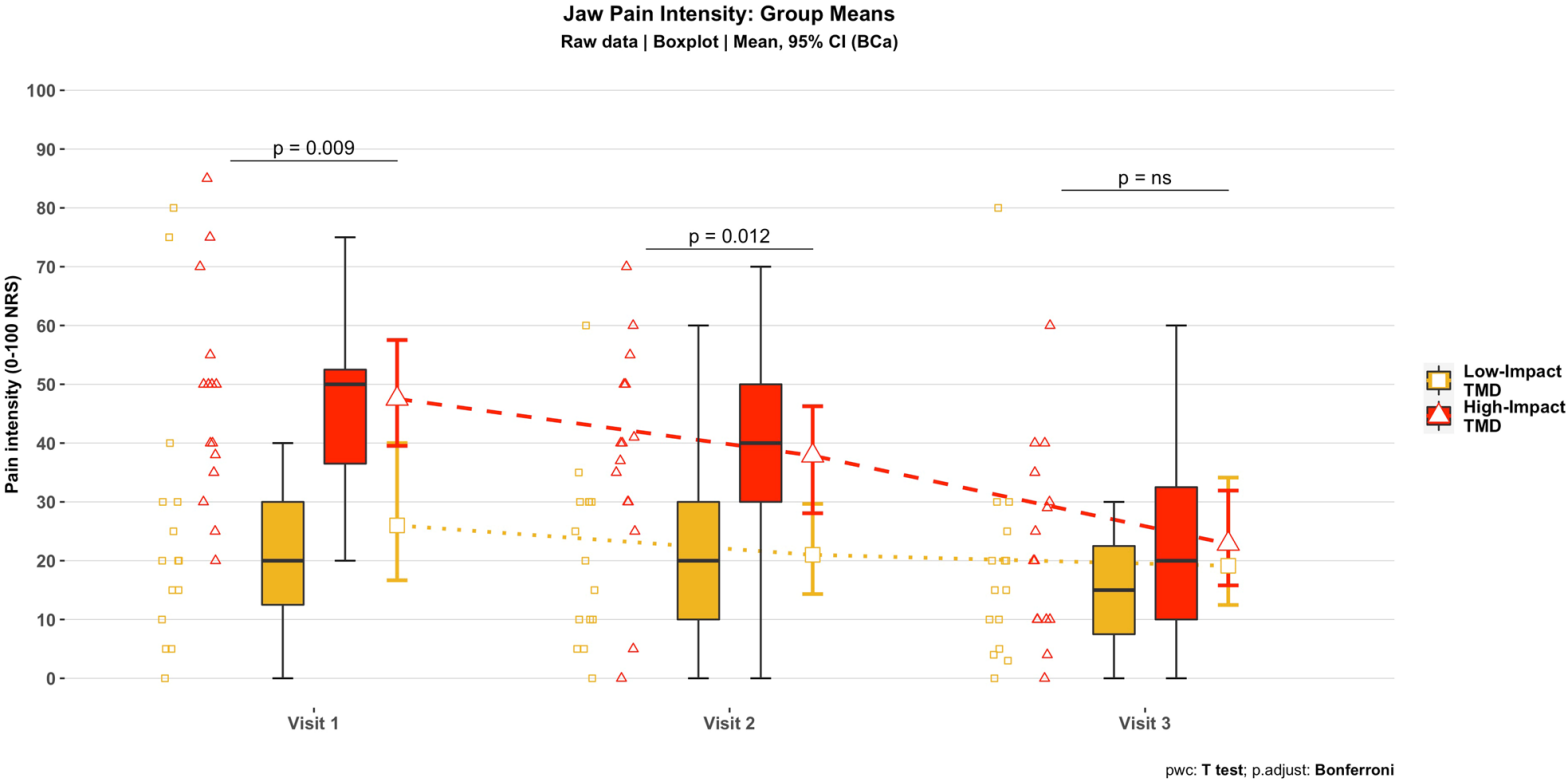
Current jaw pain intensity over time: Group means. Symbols on the left represent raw data points per participant; boxplots show median and interquartile range. Group means are shown with error bars representing their respective bias-corrected and accelerated (BCa) bootstrapped 95% confidence intervals.

Results from the linear mixed model revealed a significant group and visit interaction (F [2, 56] = 5.45, *p* = 0.007). Between-group comparisons by visit (Bonferroni-corrected *p* value cut-off = 0.017) showed significantly greater jaw pain ratings for the high-impact TMD group at visit 1 (t [28] = − 2.80; *p* = 0.009) and visit 2 (t [28] = − 2.7; *p* = 0.012) but no different than the low-impact TMD group at visit 3 (t [28] = − 0.57, *p* = 0.573) (Fig. 2).

### PPT means over time

**Figure 3:**
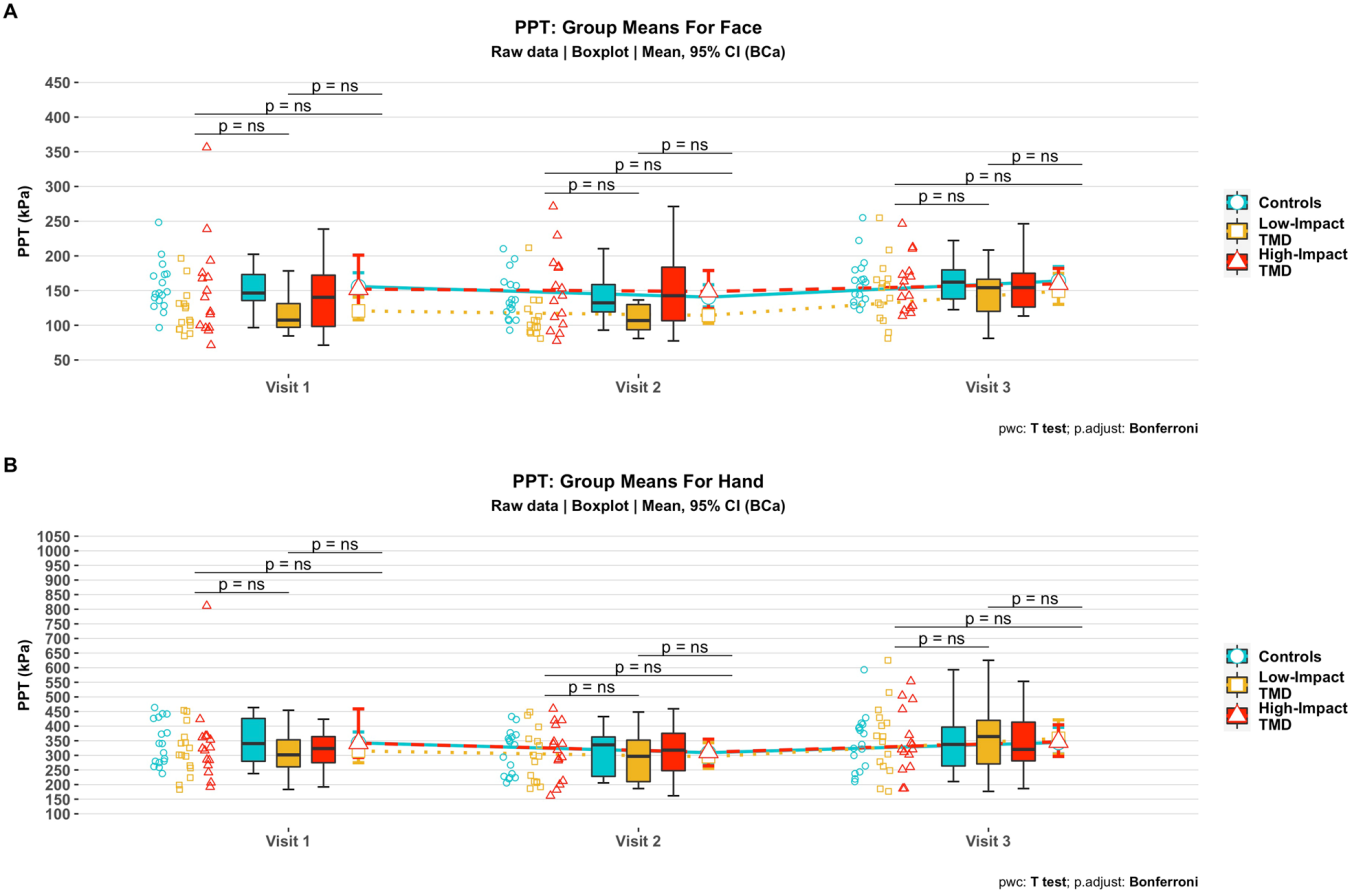
Pressure pain thresholds in the face (**A**) and hand (**B**) over time: group means. Symbols on the left represent raw data points per participant; boxplots show median and interquartile range. Group means are shown with error bars representing their respective bias-corrected and accelerated (BCa) bootstrapped 95% confidence intervals.

Regarding PPT in the face, the linear mixed model revealed a significant main effect for visit (F [2, 92] = 11.05; *p* value < 0.001), but the main effect for group was not significant (*p* = 0.131). The “group” and “visit” interaction effect was also not significant (*p* = 0.221) (Fig. 3A).

Similar findings were observed for PPT of the hand (Table 2). The main effect for visit was significant (F [2, 92] = 4.41; *p* value = 0.015) but not for the main effect for group across all visits (*p* = 0.930) nor the interaction group × visit (*p* value = 0.803) (Fig. 3B).

**Table 2:** Repeated clinical and experimental pain measures for each visit.

	Controls (n = 17)	Low-impact TMD (n = 15)	High-impact TMD (n = 15)
**Pain intensity (0**–**100 NRS)**			
Visit 1	–	26.0 (23.5)	47.5 (18.3)
Visit 2	–	21.0 (15.6)	37.9 (18.8)
Visit 3	–	19.1 (19.3)	22.9 (16.4)
**Face PPT (kPa)**			
Visit 1	155.8 (35.5)	120.7 (32.4)	152.1 (72.4)
Visit 2	140.6 (33.4)	114.3 (32.6)	148.8 (55.2)
Visit 3	163.9 (34.9)	149.2 (45.3)	159.7 (39.4)
**Hand PPT (kPa)**			
Visit 1	342.8 (76.0)	314.9 (83.9)	341.8 (144.8)
Visit 2	309.2 (76.2)	295.5 (89.1)	311.0 (92.5)
Visit 3	343.8 (75.6)	358.0 (122.1)	343.4 (111.5)

Results are presented as mean and standard deviation.

*NRS* numerical rating scale, *PPT* pressure pain thresholds, *TMD* temporomandibular disorders.

### Associations between mean jaw pain intensity and mean PPT over time

**Figure 4:**
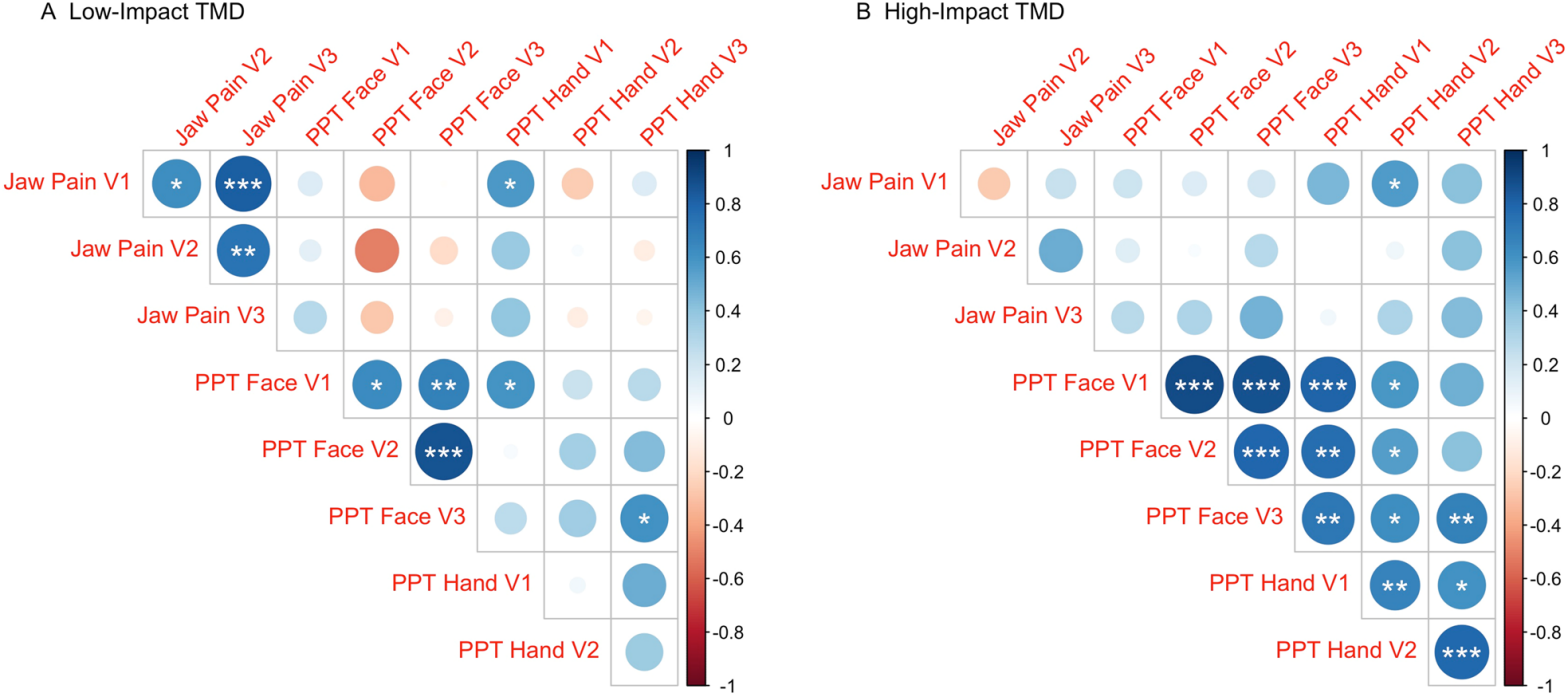
Correlograms for Pearson’s correlation coefficient in low-impact (**A**) and high-impact (**B**) TMD pain cases. The diameter and color of the circles is proportional to the correlation coefficient magnitude and sign, respectively, as shown in the color bar on the right. *p* values for correlations represented as: “*” ≤ 0.05; “**” ≤ 0.01; “***” ≤ 0.001.

Univariate correlation analyses between jaw pain intensity ratings, PPT face, and PPT hand for each of the three visits for both TMD groups are shown in Fig. 4. While the correlations between PPT and jaw pain were non-significant in both TMD groups, the low-impact TMD group displayed significant positive correlations between jaw pain ratings for all three visits, with correlation for PPT face in visit 1 and visit 3 (Fig. 4A) surviving Bonferroni correction. In contrast, the high-impact TMD group showed no significant correlations between jaw pain ratings at each visit, yet it had significant positive correlations between PPT for the face and hand across visits with several of them below Bonferroni-corrected *p* value threshold (Fig. 4B). Results from the linear model including jaw pain intensity and PPT did not reveal any significant associations between jaw pain ratings and PPT of the face (*p* = 0.968) and the hand (*p* = 0.071).

### Potential sensory testing confounders

Most potential sensory testing confounders tested (menstrual phase, caffeine intake, medication intake) did not differ between the three groups over time, nor were associated with pain-impact status or jaw pain intensity ratings for TMD groups (Supplementary Table S1). For starting time of the visit, significant positive associations were observed with jaw pain intensity ratings across all TMD participants (F [2, 21] = 6.04; *p* value = 0.009), as later starting times were associated with higher pain intensity (“6:00–10:00” vs. “14:00+”, *p* value = 0.025; “10:01–14:00” vs. “14:01 or later”, *p* value = 0.004); however when the model included pain-impact status for TMD participants plus its interaction with visits, the starting time of the visit was no longer significant (*p* value = 0.738).

## Discussion

**Figure 5:**
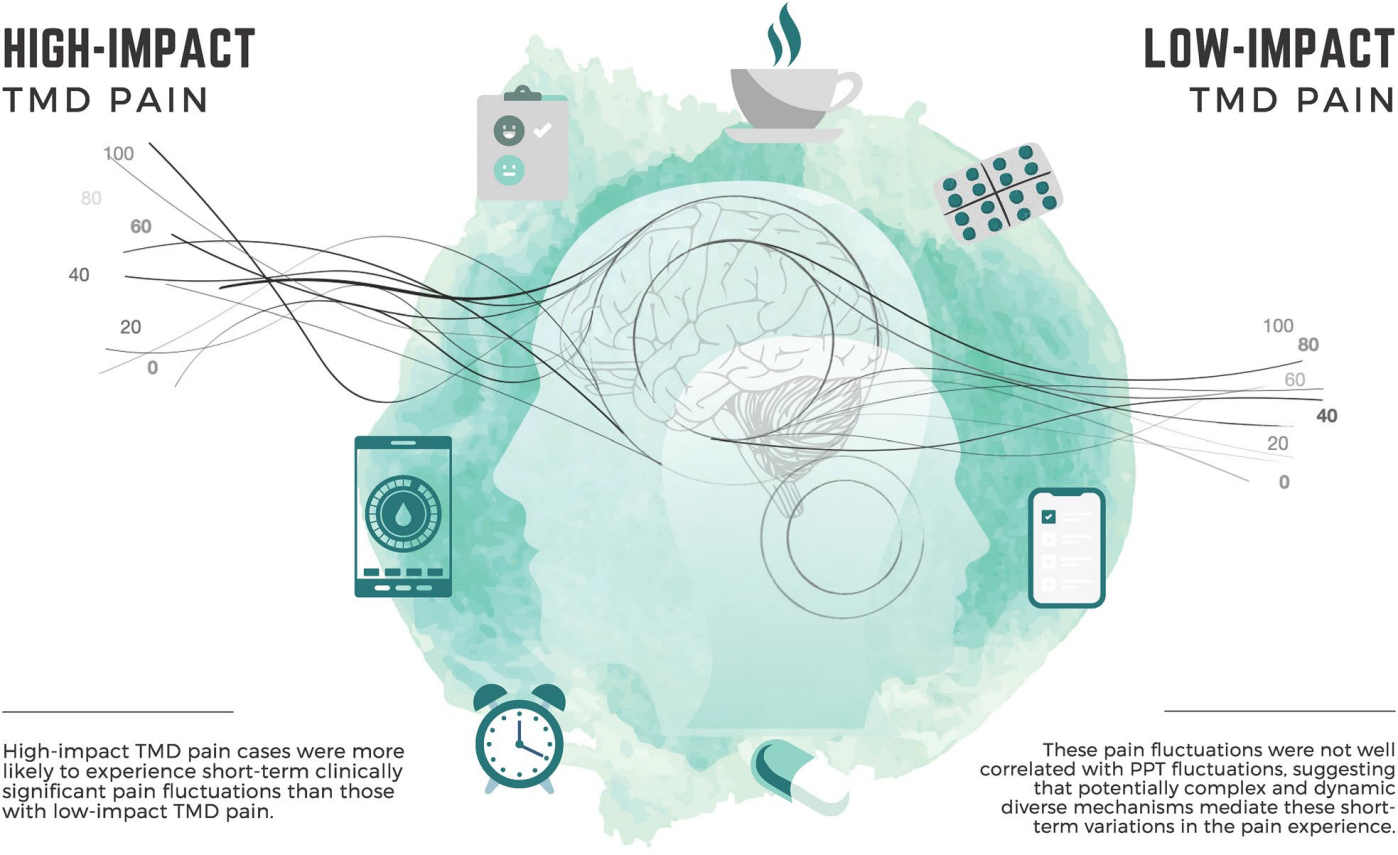
Graphical summary of the study from a conceptual illustration perspective.

The main outcomes from the present proof-of-concept study suggest that individuals with high-impact TMD pain have greater likelihood of experiencing clinically significant pain fluctuations within a short-term period than those categorized as having low-impact TMD pain. Indeed, high-impact TMD cases had 5.5 greater odds of experiencing jaw pain fluctuations relative to low-impact TMD cases, indicating that short-term jaw pain fluctuations may be at least partially associated with greater impact for TMD pain. In addition, we found that jaw pain intensity ratings were not associated with PPT when assessed over trigeminal and non-trigeminal body sites, potentially suggesting dissociated mechanisms underlying fluctuations of ongoing clinical jaw pain and evoked PPT measurements over short-term periods (Fig. 5).

Reports of pain fluctuations over time and their possible determinants have increased in numbers over the last decade to address the need to better understand the pain experience of chronic pain cases in a longitudinal fashion. Several factors have been associated with the presence of clinically significant pain fluctuations in diverse pain conditions. For example, mechanical issues such as knee and hip buckling have been associated with more short-term pain exacerbations in patients with osteoarthritis^63,64^, while psychological problems such as depression and stress have been independently associated with a higher risk of pain flares in low back pain^28^. Other factors such as poor sleep quality and heightened stress were also implicated with pain flares in qualitative studies among patients with fibromyalgia^65^. Moreover, pain fluctuations have also been associated with external factors such as weather^65,66^ and daily temperature variations^67^, smoking^25^ among other reported factors^68^. Altogether, these findings support the view that the pain experience over time is a highly complex and dynamic process, and teasing apart which factors contribute most to pain fluctuations for a particular individual may assist in developing personalized treatment strategies aimed at reducing those fluctuations and their deleterious effects to global health self-perception.

Several studies have recognized disability (i.e., impairment in daily activities) as not only an important consequence^69^ of but also a contributor to pain fluctuations in different pain conditions^24,70^. For instance, it was found that in patients with rheumatoid arthritis the functional disability assessed with the Health Assessment Questionnaire–Disability Index (HAQ-DI) at baseline was independently associated with an increased risk of pain flares over a 12 months period^24,70^. The GCPS is a widely used instrument to assess the global severity of chronic pain based on its related disability^29^, and it is probably one of the most frequently used among orofacial pain disorders^71–74^. To the best of our knowledge, no previous study investigated the influence of pain-related impact status on short-term jaw pain fluctuations in TMD cases. As stated previously, our results suggest that high-impact TMD pain cases were more likely to experience pain fluctuations than those with low-impact pain observed over a < 21 days period. One possible explanation is that high-impact TMD pain patients present a more “active” state of the disease. TMD is a heterogenous group of muscular and TMJ disorders, which can include inflammation especially when the TMJ is affected. Although our TMD case definition is based on myofascial pain, the presence or absence of arthralgia was not an exclusion criterion, therefore one cannot rule out that different pathophysiological mechanisms that could be related to arthralgia^35,75^, such as for example inflammation (e.g., due to arthritis)^76–78^, could lead to jaw pain exacerbations. Against this possibility is the fact that the presence of arthralgia was equally present among TMD cases (86.7% in each TMD group). In addition, even though patient pain-impact status were determined prior to the assessment of jaw pain fluctuations, we cannot exclude that chronic and frequent pain fluctuations prior to study enrollment is related to TMD cases reporting greater disability related to jaw pain thus leading them to be categorized as high-impact TMD cases. Future studies will need to address the direction of this association more closely, possibly including different diagnostic subgroups or profiles (i.e., only myofascial pain, only arthralgia, combined myofascial pain and arthralgia, etc.), as their underpinnings and outcomes may differ^79–81^.

Experimental pain assessed by PPT over the face and hand did not show significant associations with clinical jaw pain ratings reported by TMD cases, which is consistent with observations of previous studies^43^. Similarly, PPT changes over time could not predict the incidence of new onset TMD in the Prospective Evaluation and Risk Assessment (OPPERA) study, likely the largest prospective investigation of TMD incidence published to date^42^. This lack of association between clinical pain ratings and PPT was also demonstrated for other pain conditions such as fibromyalgia, whiplash, and low back pain^82–85^, perhaps suggesting that ongoing clinical pain and evoked pain assessed with PPT likely involve different contribution of peripheral and central pain mechanisms^82^. However, this remains as an educated conjecture and it needs to be interpreted cautiously within the complexity of the chronic pain conundrum, as for example a recent study found that local PPT only explained a 9% variance in resting (i.e., not provoked) pain ratings^85^. In addition, there is evidence pointing towards other sources of variability such as the difference between static (e.g., pain threshold) and dynamic (e.g., temporal summation) pain sensitivity ratings, and between movement-evoked pain and traditional clinical pain measures^85–87^, possibly suggesting different underlying mechanisms. In that way, the assessment of pain in TMD in a more exhaustive fashion, also including dynamic tests and motor tasks such as talking, yawning, or chewing becomes highly pertinent in the study of pain fluctuations.

Remarkably, we did not observe a statistically significant difference in PPT between cases and controls, despite low-impact TMD cases presenting numerically lower mean PPT in the face in visits 1 and 2. This finding is in contrast to a body of literature reporting decreased PPT over the masseter muscles in TMD cases when compared to controls^88–90^, however this discrepancy has also been reported previously by others^91^ and it has been recently suggested that somatosensory amplification may act as confounder for decreased PPT^92^. We observed that jaw pain ratings across visits were correlated between each other in the low-impact TMD group, while in the high-impact TMD group significant positive correlations were found between PPT across visits (both face and hand sites), which could suggest that pain-related impact status differentially contributes to the longitudinal pattern for clinical and experimental pain in each of these TMD groups. Future studies investigating the association between pain fluctuations using methods that can probe central neural pain mechanisms, such as endogenous pain modulation and neuroimaging may help advance our understanding of the role of pain fluctuations in chronic TMD.

The GCPS is thought to characterize patients suffering from diverse pain conditions and categorize them in a way that improves pain management leading to more efficient care^32,59^. One potential treatment strategy arising from the classification of TMD patients based on pain-related impact is that treatment strategies focused on self-care recommendations in non-specialist settings may be more appropriate for “functional” TMD patients (low-impact pain), whereas “dysfunctional” TMD patients (high-impact pain) may be better served by adding psychological interventions such as cognitive behavioral therapy in specialist care centers^32,33^, given their increased report of psychosocial issues^93,94^. Interestingly, we did not observe psychosocial scores differences between high- and low-impact TMD pain groups (Table 1), as one could originally expect. Whereas both TMD groups presented a higher number of comorbidities and painful body sites, somatization, depressive symptoms, oral habits, jaw limitation and poorer sleep quality than healthy controls, psychosocial measures did not differ between TMD groups. A possible explanation might be the existence of other subgroups and pain disability gradients with TMD cases, as past research observed that differences in psychosocial scores were more pronounced between painful TMD patients with no disability (GCPS grades I and II with no disability points) and high disability (GCPS grades III and IV with 3–6 disability points) when they were categorized into three groups (i.e., no disability, low disability, high disability)^33,95^. Future studies are needed to determine the presence of different subgroups of TMD cases, possibly using the recently proposed revision of the GCPS (GCPS-R)^96^, where an additional category in between low- and high- impact pain, such as “bothersome” (moderate to severe pain intensity with lower interference with life activities as per the GCPS-R), could prove to be more specific to capture clinical differences.

This study presents some limitations to be considered. First, it is based on secondary analyses of a parent study that was designed to assess outcomes related to somatosensory characteristics and neuroimaging, so the sample size included was not based on a priori power analysis to detect the effect of the statistical tests at the pre-established significance level of 0.05 for the primary outcomes reported herein. Chronic pain is a complex phenomenon with inherent within and between person high variability, influenced by numerous internal and external variables^9,10^. In adult musculoskeletal conditions, pain fluctuations are recognized as a complex, multi-layered, whole-body experience events that affect quality of life through different angles that are not only limited to pain and are unique for each individual^10,97–99^. Hence, the limited sample size in this study is unlikely to capture the full complexity of pain fluctuations, and larger studies with increased sample size and experimental visits are encouraged. Second, as only females were included given the higher prevalence of TMD in females, these results might not be generalizable to male TMD cases. Third, a denser sampling of jaw pain ratings would improve the assessment of within-person short-term variability for the included outcomes. Micro-longitudinal studies with daily assessments that may include two or more intra-day measurements can provide more insight about both day-to-day and intra-day symptoms fluctuations in TMD patients. One option is the use of electronic diaries as they can provide measurements of short-term pain changes assessed in a natural environment rather than in experimental settings^100,101^, thus allowing more adequate “real-life” pain fluctuations estimation and identification of their determinants. Moreover, as mentioned earlier, the use of dynamic sensory tests and motor tasks in addition to static sensory measures is also encouraged.

In conclusion, results from this proof-of-concept study suggest that high-impact TMD pain cases are more likely to experience short-term clinically significant pain fluctuations than those with low-impact TMD pain. These pain fluctuations were not well correlated with PPT fluctuations, suggesting that potentially complex and dynamic diverse mechanisms mediate these short-term variations in the pain experience. Due to the nature of the study, caution is warranted when interpreting these results. Future studies are needed to better understand the role of pain-related impact level and other possible determinants for short-term jaw pain fluctuations among TMD cases, with the goal of developing targeted treatment strategies to reduce their occurrence leading to improved patient perception of clinical pain management outcomes.

## Supplementary Information

Supplementary Table S1.

## References

[1] Harris RE, Williams DA, McLean SA, Sen A, Hufford M, Gendreau RM, Gracely RH, Clauw DJ (2005). Characterization and consequences of pain variability in individuals with fibromyalgia. Arthritis Rheum..

[2] Hutchings A, Calloway M, Choy E, Hooper M, Hunter DJ, Jordan JM, Zhang Y, Baser O, Long S, Palmer L (2007). The longitudinal examination of arthritis pain (LEAP) study: Relationships between weekly fluctuations in patient-rated joint pain and other health outcomes. J. Rheumatol..

[3] Glette M, Stiles TC, Borchgrevink PC, Landmark T (2020). The natural course of chronic pain in a general population: Stability and change in an eight-wave longitudinal study over four years (the HUNT Pain Study). J. Pain.

[4] Radojcic MR, Arden NK, Yang X, Strauss VY, Birrell F, Cooper C, Kluzek S (2020). Pain trajectory defines knee osteoarthritis subgroups: A prospective observational study. Pain.

[5] Parry E, Ogollah R, Peat G (2019). 'Acute flare-ups' in patients with, or at high risk of, knee osteoarthritis: A daily diary study with case-crossover analysis. Osteoarthr. Cartil..

[6] Kerns RD, Finn P, Haythornthwaite J (1988). Self-monitored pain intensity: Psychometric properties and clinical utility. J. Behav. Med..

[7] Schneider S, Junghaenel DU, Keefe FJ, Schwartz JE, Stone AA, Broderick JE (2012). Individual differences in the day-to-day variability of pain, fatigue, and well-being in patients with rheumatic disease: Associations with psychological variables. Pain.

[8] Kerns RD, Haythornthwaite JA (1988). Depression among chronic pain patients: Cognitive-behavioral analysis and effect on rehabilitation outcome. J. Consult. Clin. Psychol..

[9] Costa N, Smits EJ, Kasza J, Salomoni SE, Ferreira M, Hodges PW (2021). Low back pain flares: How do they differ from an increase in pain?. Clin. J. Pain.

[10] Khanom S, McDonagh JE, Briggs M, Bakir E, McBeth J (2020). Adolescents' experiences of fluctuating pain in musculoskeletal disorders: A qualitative systematic review and thematic synthesis. BMC Musculoskelet. Disord..

[11] Carriere JS, Lazaridou A, Martel MO, Cornelius M, Campbell C, Smith M, Haythornthwaite JA, Edwards RR (2020). The moderating role of pain catastrophizing on the relationship between partner support and pain intensity: A daily diary study in patients with knee osteoarthritis. J. Behav. Med..

[12] Schiffman E, Ohrbach R, Truelove E, Look J, Anderson G, Goulet JP, List T, Svensson P, Gonzalez Y, Lobbezoo F, Michelotti A, Brooks SL, Ceusters W, Drangsholt M, Ettlin D, Gaul C, Goldberg LJ, Haythornthwaite JA, Hollender L, Jensen R, John MT, De Laat A, de Leeuw R, Maixner W, van der Meulen M, Murray GM, Nixdorf DR, Palla S, Petersson A, Pionchon P, Smith B, Visscher CM, Zakrzewska J, Dworkin SF (2014). Diagnostic criteria for temporomandibular disorders (DC/TMD) for clinical and research applications: recommendations of the International RDC/TMD consortium network* and orofacial pain special interest groupdagger. J. Oral Facial Pain Headache..

[13] Slade GD, Ohrbach R, Greenspan JD, Fillingim RB, Bair E, Sanders AE, Dubner R, Diatchenko L, Meloto CB, Smith S, Maixner W (2016). Painful temporomandibular disorder: Decade of discovery from OPPERA studies. J. Dent. Res..

[14] Cioffi I, Farella M, Chiodini P, Ammendola L, Capuozzo R, Klain C, Vollaro S, Michelotti A (2017). Effect of weather on temporal pain patterns in patients with temporomandibular disorders and migraine. J. Oral Rehabil..

[15] Sherman JJ, LeResche L, Mancl LA, Huggins K, Sage JC, Dworkin SF (2005). Cyclic effects on experimental pain response in women with temporomandibular disorders. J. Orofac. Pain.

[16] Dworkin RH, Turk DC, Wyrwich KW, Beaton D, Cleeland CS, Farrar JT, Haythornthwaite JA, Jensen MP, Kerns RD, Ader DN, Brandenburg N, Burke LB, Cella D, Chandler J, Cowan P, Dimitrova R, Dionne R, Hertz S, Jadad AR, Katz NP, Kehlet H, Kramer LD, Manning DC, McCormick C, McDermott MP, McQuay HJ, Patel S, Porter L, Quessy S, Rappaport BA, Rauschkolb C, Revicki DA, Rothman M, Schmader KE, Stacey BR, Stauffer JW, von Stein T, White RE, Witter J, Zavisic S (2008). Interpreting the clinical importance of treatment outcomes in chronic pain clinical trials: IMMPACT recommendations. J. Pain.

[17] Smith SM, Dworkin RH, Turk DC, McDermott MP, Eccleston C, Farrar JT, Rowbotham MC, Bhagwagar Z, Burke LB, Cowan P, Ellenberg SS, Evans SR, Freeman RL, Garrison LP, Iyengar S, Jadad A, Jensen MP, Junor R, Kamp C, Katz NP, Kesslak JP, Kopecky EA, Lissin D, Markman JD, Mease PJ, O'Connor AB, Patel KV, Raja SN, Sampaio C, Schoenfeld D, Singh J, Steigerwald I, Strand V, Tive LA, Tobias J, Wasan AD, Wilson HD (2020). Interpretation of chronic pain clinical trial outcomes: IMMPACT recommended considerations. Pain.

[18] Glaros AG, Williams K, Lausten L (2008). Diurnal variation in pain reports in temporomandibular disorder patients and control subjects. J. Orofac. Pain.

[19] Abou-Atme YS, Melis M, Zawawi KH, Cottogno L (2007). Five-year follow-up of temporomandibular disorders and other musculoskeletal symptoms in dental students. Minerva Stomatol..

[20] Nilsson IM, List T, Drangsholt M (2007). Incidence and temporal patterns of temporomandibular disorder pain among Swedish adolescents. J. Orofac. Pain.

[21] Vilanova LS, Goncalves TM, Meirelles L, Garcia RC (2015). Hormonal fluctuations intensify temporomandibular disorder pain without impairing masticatory function. Int. J. Prosthodont..

[22] Robinson RC, Garofalo JP, Gatchel RJ (2006). Decreases in cortisol variability between treated and untreated jaw pain patients. J. Appl. Biobehav. Res..

[23] Turner JA, Mancl L, Huggins KH, Sherman JJ, Lentz G, LeResche L (2011). Targeting temporomandibular disorder pain treatment to hormonal fluctuations: A randomized clinical trial. Pain.

[24] Bechman K, Tweehuysen L, Garrood T, Scott DL, Cope AP, Galloway JB, Ma MHY (2018). Flares in rheumatoid arthritis patients with low disease activity: Predictability and association with worse clinical outcomes. J. Rheumatol..

[25] Oh YJ, Moon KW (2020). Predictors of flares in patients with rheumatoid arthritis who exhibit low disease activity: A nationwide cohort study. J. Clin. Med..

[26] Trouvin AP, Marty M, Goupille P, Perrot S (2019). Determinants of daily pain trajectories and relationship with pain acceptability in hip and knee osteoarthritis: A national prospective cohort study on 886 patients. Joint Bone Spine.

[27] Wieczorek M, Rotonda C, Coste J, Pouchot J, Saraux A, Guillemin F, Rat AC (2020). Trajectory analysis combining pain and physical function in individuals with knee and hip osteoarthritis: Results from the French KHOALA cohort. Rheumatology.

[28] Suri P, Rainville J, de Schepper E, Martha J, Hartigan C, Hunter D (2018). Do physical activities trigger flare-ups during an acute low back pain episode? A longitudinal case-crossover feasibility study. Spine.

[29] Von Korff M, Ormel J, Keefe FJ, Dworkin SF (1992). Grading the severity of chronic pain. Pain.

[30] Dworkin SF, Huggins KH, Wilson L, Mancl L, Turner J, Massoth D, LeResche L, Truelove E (2002). A randomized clinical trial using research diagnostic criteria for temporomandibular disorders-axis II to target clinic cases for a tailored self-care TMD treatment program. J. Orofac. Pain.

[31] Breckons M, Bissett SM, Exley C, Araujo-Soares V, Durham J (2017). Care pathways in persistent orofacial pain: Qualitative evidence from the DEEP study. JDR Clin. Transl. Res..

[32] Durham J, Shen J, Breckons M, Steele JG, Araujo-Soares V, Exley C, Vale L (2016). Healthcare cost and impact of persistent orofacial pain: The DEEP study cohort. J. Dent. Res..

[33] Kotiranta U, Suvinen T, Kauko T, Le Bell Y, Kemppainen P, Suni J, Forssell H (2015). Subtyping patients with temporomandibular disorders in a primary health care setting on the basis of the research diagnostic criteria for temporomandibular disorders axis II pain-related disability: A step toward tailored treatment planning?. J. Oral Facial Pain Headache.

[34] Manfredini D, Favero L, Gregorini G, Cocilovo F, Guarda-Nardini L (2013). Natural course of temporomandibular disorders with low pain-related impairment: A 2-to-3-year follow-up study. J. Oral Rehabil..

[35] Harper DE, Schrepf A, Clauw DJ (2016). Pain mechanisms and centralized pain in temporomandibular disorders. J. Dent. Res..

[36] Moana-Filho EJ, Herrero Babiloni A, Theis-Mahon NR (2018). Endogenous pain modulation in chronic orofacial pain: A systematic review and meta-analysis. Pain.

[37] Breckons M, Shen J, Bunga J, Vale L, Durham J (2018). DEEP study: Indirect and out-of-pocket costs of persistent orofacial pain. J. Dent. Res..

[38] Rolke R, Baron R, Maier C, Tolle TR, Treede RD, Beyer A, Binder A, Birbaumer N, Birklein F, Botefur IC, Braune S, Flor H, Huge V, Klug R, Landwehrmeyer GB, Magerl W, Maihofner C, Rolko C, Schaub C, Scherens A, Sprenger T, Valet M, Wasserka B (2006). Quantitative sensory testing in the German research network on neuropathic pain (DFNS): Standardized protocol and reference values. Pain.

[39] Treede RD, Rolke R, Andrews K, Magerl W (2002). Pain elicited by blunt pressure: Neurobiological basis and clinical relevance. Pain.

[40] Castien RF, van der Wouden JC, De Hertogh W (2018). Pressure pain thresholds over the cranio-cervical region in headache: A systematic review and meta-analysis. J. Headache Pain.

[41] Svensson P, Baad-Hansen L, Pigg M, List T, Eliav E, Ettlin D, Michelotti A, Tsukiyama Y, Matsuka Y, Jaaskelainen SK, Essick G, Greenspan JD, Drangsholt M (2011). Guidelines and recommendations for assessment of somatosensory function in oro-facial pain conditions—A taskforce report. J. Oral Rehabil..

[42] Slade GD, Sanders AE, Ohrbach R, Fillingim RB, Dubner R, Gracely RH, Bair E, Maixner W, Greenspan JD (2014). Pressure pain thresholds fluctuate with, but do not usefully predict, the clinical course of painful temporomandibular disorder. Pain.

[43] Sanches ML, Juliano Y, Novo NF, Guimaraes AS, Rodrigues Conti PC, Alonso LG (2015). Correlation between pressure pain threshold and pain intensity in patients with temporomandibular disorders who are compliant or non-compliant with conservative treatment. Oral Surg. Oral Med. Oral Pathol. Oral Radiol..

[44] Moana-Filho EJ, Herrero BA (2019). Endogenous pain modulation in chronic temporomandibular disorders: Derivation of pain modulation profiles and assessment of its relationship with clinical characteristics. J. Oral Rehabil..

[45] Moana-Filho EJ, Herrero Babiloni A, Nisley A (2019). Endogenous pain modulation assessed with offset analgesia is not impaired in chronic temporomandibular disorder pain patients. J. Oral Rehabil..

[46] Donnell A, T DN, Lawrence M, Gupta V, Zieba T, Truong DQ, Bikson M, Datta A, Bellile E, DaSilva AF,  (2015). High-definition and non-invasive brain modulation of pain and motor dysfunction in chronic TMD. Brain Stimul..

[47] Raphael KG, Janal MN, Sirois DA, Dubrovsky B, Wigren PE, Klausner JJ, Krieger AC, Lavigne GJ (2013). Masticatory muscle sleep background electromyographic activity is elevated in myofascial temporomandibular disorder patients. J. Oral Rehabil..

[48] Vidor LP, Torres IL, Custodio de Souza IC, Fregni F, Caumo W (2013). Analgesic and sedative effects of melatonin in temporomandibular disorders: A double-blind, randomized, parallel-group, placebo-controlled study. J. Pain Sympt. Manag..

[49] LeResche L (1997). Epidemiology of temporomandibular disorders: Implications for the investigation of etiologic factors. Crit. Rev. Oral Biol. Med..

[50] Miller VE, Poole C, Golightly Y, Barrett D, Chen DG, Ohrbach R, Greenspan JD, Fillingim RB, Slade GD (2019). Characteristics associated with high-impact pain in people with temporomandibular disorder: A cross-sectional study. J. Pain..

[51] Cohen S, Kamarck T, Mermelstein R (1983). A global measure of perceived stress. J. Health Soc. Behav..

[52] Buysse DJ, Reynolds CF, Monk TH, Berman SR, Kupfer DJ (1989). The Pittsburgh Sleep Quality Index: A new instrument for psychiatric practice and research. Psychiatry Res..

[53] Turk DC, Melzack R, Guilford The (2011). The measurement of pain and the assessment of people experiencing pain. Handbook of pain assessment.

[54] Lewis GN, Rice DA, McNair PJ (2012). Conditioned pain modulation in populations with chronic pain: A systematic review and meta-analysis. J Pain.

[55] Riley JL, Robinson ME, Wise EA, Price DD (1999). A meta-analytic review of pain perception across the menstrual cycle. Pain.

[56] Sawynok J (2011). Caffeine and pain. Pain.

[57] Harden RN, Weinland SR, Remble TA, Houle TT, Colio S, Steedman S, Kee WG (2005). Medication Quantification Scale Version III: update in medication classes and revised detriment weights by survey of American Pain Society Physicians. J. Pain.

[58] Penlington C, Araujo-Soares V, Durham J (2020). Predicting persistent orofacial pain: The role of illness perceptions, anxiety, and depression. JDR Clin. Transl. Res..

[59] Visscher CM, Baad-Hansen L, Durham J, Goulet JP, Michelotti A, Roldan Barraza C, Haggman-Henrikson B, Ekberg E, Raphael KG (2018). Benefits of implementing pain-related disability and psychological assessment in dental practice for patients with temporomandibular pain and other oral health conditions. J. Am. Dent. Assoc..

[60] Pitcher MH, Von Korff M, Bushnell MC, Porter L (2019). Prevalence and profile of high-impact chronic pain in the United States. J. Pain.

[61] de Koning EJ, Timmermans EJ, van Schoor NM, Stubbs B, van den Kommer TN, Dennison EM, Limongi F, Castell MV, Edwards MH, Queipo R, Cooper C, Siviero P, van der Pas S, Pedersen NL, Sanchez-Martinez M, Deeg DJH, Denkinger MD (2018). Within-person pain variability and mental health in older adults with osteoarthritis: An analysis across 6 European Cohorts. J. Pain.

[62] Parry E, Ogollah R, Peat G (2017). Significant pain variability in persons with, or at high risk of, knee osteoarthritis: Preliminary investigation based on secondary analysis of cohort data. BMC Musculoskelet. Disord..

[63] Fu K, Makovey J, Metcalf B, Bennell K, Zhang Y, Asher R, Robbins S, Deveza L, Hunter DJ (2019). Role of hip injury and giving way in pain exacerbation in hip osteoarthritis: An internet-based case-crossover study. Arthritis Care Res..

[64] Zobel I, Erfani T, Bennell KL, Makovey J, Metcalf B, Chen JS, March L, Zhang Y, Eckstein F, Hunter DJ (2016). Relationship of buckling and knee injury to pain exacerbation in knee osteoarthritis: A web-based case-crossover study. Interact. J. Med. Res..

[65] Vincent A, Whipple MO, Rhudy LM (2016). Fibromyalgia flares: A qualitative analysis. Pain Med..

[66] Li J, Yu T, Javed I, Siddagunta C, Pakpahan R, Langston ME, Dennis LK, Kingfield DM, Moore DJ, Andriole GL, Lai HH, Colditz GA, Sutcliffe S, Network MR (2020). Does weather trigger urologic chronic pelvic pain syndrome flares? A case-crossover analysis in the multidisciplinary approach to the study of the chronic pelvic pain research network. Neurourol. Urodyn..

[67] Fu K, Metcalf B, Bennell KL, Zhang Y, Deveza LA, Robbins SR, Ferreira ML, Hunter DJ (2020). Association of weather factors with the risk of pain exacerbations in people with hip osteoarthritis. Scandinav. J. Rheumatol..

[68] Costa N, Hodges PW, Ferreira ML, Makovey J, Setchell J (2019). What triggers an LBP flare? A content analysis of individuals’ perspectives. Pain Med..

[69] Christopher-Stine L, Wan GJ, Kelly W, McGowan M, Bostic R, Reed ML (2020). Patient-reported dermatomyositis and polymyositis flare symptoms are associated with disability, productivity loss, and health care resource use. J. Manag. Care Spec. Pharm..

[70] Saleem B, Brown AK, Quinn M, Karim Z, Hensor EM, Conaghan P, Peterfy C, Wakefield RJ, Emery P (2012). Can flare be predicted in DMARD treated RA patients in remission, and is it important? A cohort study. Ann. Rheum. Dis..

[71] Brailo V, Zakrzewska JM (2015). Grading the intensity of nondental orofacial pain: Identification of cutoff points for mild, moderate, and severe pain. J. Pain Res..

[72] Chung JW, Kim JH, Kim HD, Kho HS, Kim YK, Chung SC (2004). Chronic orofacial pain among Korean elders: Prevalence, and impact using the graded chronic pain scale. Pain.

[73] Cioffi I, Perrotta S, Ammendola L, Cimino R, Vollaro S, Paduano S, Michelotti A (2014). Social impairment of individuals suffering from different types of chronic orofacial pain. Prog. Orthod..

[74] De La Torre CG, Camara-Souza MB, Munoz Lora VRM, Guarda-Nardini L, Conti PCR, Rodrigues Garcia RM, Del Bel Cury AA, Manfredini D (2018). Prevalence of psychosocial impairment in temporomandibular disorder patients: A systematic review. J. Oral Rehabil..

[75] Palmer J, Durham J (2021). Temporomandibular disorders. BJA Educ..

[76] Cairns BE (2010). Pathophysiology of TMD pain–basic mechanisms and their implications for pharmacotherapy. J. Oral Rehabil..

[77] Kothari SF, Baad-Hansen L, Hansen LB, Bang N, Sorensen LH, Eskildsen HW, Svensson P (2016). Pain profiling of patients with temporomandibular joint arthralgia and osteoarthritis diagnosed with different imaging techniques. J. Headache Pain.

[78] Takeuchi Y, Zeredo JL, Fujiyama R, Amagasa T, Toda K (2004). Effects of experimentally induced inflammation on temporomandibular joint nociceptors in rats. Neurosci. Lett..

[79] Cao Y, Yap AU, Lei J, Zhang MJ, Fu KY (2021). Subtypes of acute and chronic temporomandibular disorders: Their relation to psychological and sleep impairments. Oral Dis..

[80] Yap AU, Cao Y, Zhang MJ, Lei J, Fu KY (2021). Comparison of emotional disturbance, sleep, and life quality in adult patients with painful temporomandibular disorders of different origins. Clin. Oral Invest..

[81] Yap AU, Cao Y, Zhang MJ, Lei J, Fu KY (2021). Temporomandibular disorder severity and diagnostic groups: Their associations with sleep quality and impairments. Sleep Med..

[82] Kamper SJ, Maher CG, Hush JM, Pedler A, Sterling M (2011). Relationship between pressure pain thresholds and pain ratings in patients with whiplash-associated disorders. Clin. J. Pain.

[83] Laursen BS, Bajaj P, Olesen AS, Delmar C, Arendt-Nielsen L (2005). Health related quality of life and quantitative pain measurement in females with chronic non-malignant pain. Eur. J. Pain.

[84] Palsson TS, Christensen SWM, De Martino E, Graven-Nielsen T (2021). Pain and disability in low back pain can be reduced despite no significant improvements in mechanistic pain biomarkers. Clin. J. Pain.

[85] Simon CB, Lentz TA, Ellis L, Bishop MD, Fillingim RB, Riley JL, George SZ (2021). Static and dynamic pain sensitivity in adults with persistent low back pain: comparison to healthy controls and associations with movement-evoked pain versus traditional clinical pain measures. Clin. J. Pain.

[86] Coronado RA, Simon CB, Valencia C, Parr JJ, Borsa PA, George SZ (2014). Suprathreshold heat pain response predicts activity-related pain, but not rest-related pain, in an exercise-induced injury model. PloS One.

[87] Rakel BA, Blodgett NP, Zimmerman BM, Logsden-Sackett N, Clark C, Noiseux N, Callaghan J, Herr K, Geasland K, Yang X, Sluka KA (2012). Predictors of postoperative movement and resting pain following total knee replacement. Pain.

[88] Carlson CR, Reid KI, Curran SL, Studts J, Okeson JP, Falace D, Nitz A, Bertrand PM (1998). Psychological and physiological parameters of masticatory muscle pain. Pain.

[89] Greenspan JD, Slade GD, Bair E, Dubner R, Fillingim RB, Ohrbach R, Knott C, Mulkey F, Rothwell R, Maixner W (2011). Pain sensitivity risk factors for chronic TMD: Descriptive data and empirically identified domains from the OPPERA case control study. J. Pain.

[90] Svensson P, List T, Hector G (2001). Analysis of stimulus-evoked pain in patients with myofascial temporomandibular pain disorders. Pain.

[91] Quartana PJ, Finan PH, Smith MT (2015). Evidence for sustained mechanical pain sensitization in women with chronic temporomandibular disorder versus healthy female participants. J. Pain.

[92] Spano VE, Imbriglio TV, Ho KCJ, Chow JCF, Cioffi I (2021). Increased somatosensory amplification is associated with decreased pressure pain thresholds at both trigeminal and extra-trigeminal locations in healthy individuals. J. Oral Rehabil..

[93] De la Torre CG, Bonjardim LR, Poluha RL, Carvalho Soares FF, Guarda-Nardini L, Conti PR, Manfredini D (2020). Correlation between physical and psychosocial findings in a population of temporomandibular disorder patients. Int. J. Prosthodont..

[94] Manfredini D, Winocur E, Ahlberg J, Guarda-Nardini L, Lobbezoo F (2010). Psychosocial impairment in temporomandibular disorders patients. RDC/TMD axis II findings from a multicentre study. J. Dentistry.

[95] Suvinen TI, Kemppainen P, Le Bell Y, Valjakka A, Vahlberg T, Forssell H (2013). Research Diagnostic Criteria Axis II in screening and as a part of biopsychosocial subtyping of Finnish patients with temporomandibular disorder pain. J. Orofac. Pain.

[96] Von Korff M, DeBar LL, Krebs EE, Kerns RD, Deyo RA, Keefe FJ (2020). Graded chronic pain scale revised: Mild, bothersome, and high-impact chronic pain. Pain.

[97] Hewlett S, Sanderson T, May J, Alten R, Bingham CO, Cross M, March L, Pohl C, Woodworth T, Bartlett SJ (2012). 'I'm hurting, I want to kill myself': Rheumatoid arthritis flare is more than a high joint count—An international patient perspective on flare where medical help is sought. Rheumatology.

[98] Moverley AR, Vinall-Collier KA, Helliwell PS (2015). It's not just the joints, it's the whole thing: Qualitative analysis of patients' experience of flare in psoriatic arthritis. Rheumatology.

[99] Setchell J, Costa N, Ferreira M, Makovey J, Nielsen M, Hodges PW (2017). What constitutes back pain flare? A cross sectional survey of individuals with low back pain. Scand. J. Pain.

[100] Aaron LA, Turner JA, Mancl L, Brister H, Sawchuk CN (2005). Electronic diary assessment of pain-related variables: Is reactivity a problem?. J. Pain..

[101] Piasecki TM, Hufford MR, Solhan M, Trull TJ (2007). Assessing clients in their natural environments with electronic diaries: Rationale, benefits, limitations, and barriers. Psychol. Assess..

