# Supplementary material for: Temporomandibular disorders cases with high-impact pain are more likely to experience short-term pain fluctuations
Source: Sci Rep. 2022 Jan 31;12:1657. doi: 10.1038/s41598-022-05598-w (PMC8803984; doi:10.1038/s41598-022-05598-w)
Supplement: Supplementary file 1 — Supplementary Table S1. [file 41598_2022_5598_MOESM1_ESM.docx]

Supplementary Table 1. Repeated measures assessment for sensory confounders.

|  |  |  | **Controls (n=17)** | **Low-impact TMD (n=15)** | **High-impact TMD (n=15)** |
| --- | --- | --- | --- | --- | --- |
| **Time of testing** | **Visit 1** | 6:00 to 10:00 | 1 (5.9%) | 3 (20.0%) | 1 (6.7%) |
|  |  | 10:01 to 14:00 | 7 (41.2%) | 10 (66.7%) | 0 (0.0%) |
|  |  | 14:01 and later | 9 (52.9%) | 2 (13.3%) | 14 (93.3%) |
|  | **Visit 2** | 6:00 to 10:00 | 6 (35.3%) | 4 (26.7%) | 2 (13.3%) |
|  |  | 10:01 to 14:00 | 8 (47.1%) | 10 (66.7%) | 0 (0.0%) |
|  |  | 14:01 and later | 3 (17.6%) | 1 (6.7%) | 13 (86.7%) |
|  | **Visit 3** | 6:00 to 10:00 | 5 (29.4%) | 3 (20.0%) | 5 (33.3%) |
|  |  | 10:01 to 14:00 | 8 (47.1%) | 9 (60.0%) | 9 (60.0%) |
|  |  | 14:01 and later | 4 (23.5%) | 3 (20.0%) | 1 (6.7%) |
| **Caffeine intake last 24 h** | **Visit 1** | None | 4 (23.5%) | 7 (46.7%) | 7 (46.7%) |
|  |  | Low | 7 (41.2%) | 4 (26.7%) | 4 (26.7%) |
|  |  | Moderate | 3 (17.6%) | 1 (6.7%) | 2 (13.3%) |
|  |  | High | 3 (17.6%) | 3 (20.0%) | 2 (13.3%) |
|  | **Visit 2** | None | 3 (17.6%) | 5 (33.3%) | 7 (46.7%) |
|  |  | Low | 8 (47.1%) | 5 (33.3%) | 4 (26.7%) |
|  |  | Moderate | 5 (29.4%) | 2 (13.3%) | 3 (20.0%) |
|  |  | High | 1 (5.9%) | 3 (20.0%) | 1 (6.7%) |
|  | **Visit 3** | None | 6 (35.3%) | 6 (40.0%) | 9 (60.0%) |
|  |  | Low | 5 (29.4%) | 4 (26.7%) | 5 (33.3%) |
|  |  | Moderate | 3 (17.6%) | 1 (6.7%) | 1 (6.7%) |
|  |  | High | 3 (17.6%) | 4 (26.7%) | 0 (0.0%) |
| **Menses occurrence** | **Visit 1** | Menopause | 2 (11.8%) | 2 (13.3%) | 1 (6.7%) |
|  |  | Oral contraceptives w/ menses | 3 (17.6%) | 3 (20.0%) | 1 (6.7%) |
|  |  | Oral contraceptives w/o menses | 0 (0.0%) | 1 (6.7%) | 2 (13.3%) |
|  |  | Other reason | 2 (11.8%) | 2 (13.3%) | 2 (13.3%) |
|  |  | Menses occurring | 10 (58.8%) | 7 (46.7%) | 9 (60.0%) |
|  | **Visit 2** | Menopause | 2 (11.8%) | 2 (13.3%) | 1 (6.7%) |
|  |  | Oral contraceptives w/ menses | 2 (11.8%) | 2 (13.3%) | 1 (6.7%) |
|  |  | Oral contraceptives w/o menses | 0 (0.0%) | 0 (0.0%) | 2 (13.3%) |
|  |  | Other reason | 2 (11.8%) | 3 (20.0%) | 2 (13.3%) |
|  |  | Menses occurring | 11 (64.7%) | 8 (53.3%) | 9 (60.0%) |
|  | **Visit 3** | Menopause | 2 (11.8%) | 2 (13.3%) | 1 (6.7%) |
|  |  | Oral contraceptives w/ menses | 2 (11.8%) | 2 (13.3%) | 1 (6.7%) |
|  |  | Oral contraceptives w/o menses | 0 (0.0%) | 0 (0.0%) | 2 (13.3%) |
|  |  | Other reason | 3 (17.6%) | 3 (20.0%) | 2 (13.3%) |
|  |  | Menses occurring | 10 (58.8%) | 8 (53.3%) | 9 (60.0%) |
| **Menstrual cycle phase** | **Visit 1** | No menses | 4 (23.5%) | 5 (33.3%) | 5 (33.3%) |
|  |  | Menstrual | 0 (0.0%) | 3 (20.0%) | 0 (0.0%) |
|  |  | Follicular | 2 (11.8%) | 2 (13.3%) | 5 (33.3%) |
|  |  | Periovulatory | 6 (35.3%) | 1 (6.7%) | 2 (13.3%) |
|  |  | Luteal | 5 (29.4%) | 2 (13.3%) | 1 (6.7%) |
|  |  | Premenstrual | 0 (0.0%) | 2 (13.3%) | 1 (6.7%) |
|  |  | (Missing) | 0 (0.0%) | 0 (0.0%) | 1 (6.7%) |
|  | **Visit 2** | No menses | 4 (23.5%) | 5 (33.3%) | 5 (33.3%) |
|  |  | Menstrual | 0 (0.0%) | 1 (6.7%) | 1 (6.7%) |
|  |  | Follicular | 1 (5.9%) | 3 (20.0%) | 1 (6.7%) |
|  |  | Periovulatory | 7 (41.2%) | 0 (0.0%) | 4 (26.7%) |
|  |  | Luteal | 2 (11.8%) | 3 (20.0%) | 3 (20.0%) |
|  |  | Premenstrual | 3 (17.6%) | 2 (13.3%) | 0 (0.0%) |
|  |  | (Missing) | 0 (0.0%) | 1 (6.7%) | 1 (6.7%) |
|  | **Visit 3** | No menses | 4 (23.5%) | 5 (33.3%) | 5 (33.3%) |
|  |  | Menstrual | 1 (5.9%) | 2 (13.3%) | 1 (6.7%) |
|  |  | Follicular | 1 (5.9%) | 2 (13.3%) | 1 (6.7%) |
|  |  | Periovulatory | 2 (11.8%) | 1 (6.7%) | 1 (6.7%) |
|  |  | Luteal | 7 (41.2%) | 2 (13.3%) | 6 (40.0%) |
|  |  | Premenstrual | 2 (11.8%) | 2 (13.3%) | 1 (6.7%) |
|  |  | (Missing) | 0 (0.0%) | 1 (6.7%) | 0 (0.0%) |
| **Med intake last 24 h (MQS)** | **Visit 1** |  | 0.82 (1.99) | 2.75 (4.94) | 4.72 (7.82) |
|  | **Visit 2** |  | 0.71 (1.63) | 3.42 (5.21) | 3.43 (4.27) |
|  | **Visit 3** |  | 0.71 (1.63) | 3.79 (5.08) | 1.69 (2.22) |

Results are presented as number and percentage for categorical variables and mean and standard deviation for continuous variables. Abbreviations: MQS: Medication Quantification Scale TMD: temporomandibular disorders.
